# Epigenetic Regulatory Mechanisms Associated with Infertility

**DOI:** 10.1155/2010/198709

**Published:** 2010-08-05

**Authors:** Sheroy Minocherhomji, Prochi F. Madon, Firuza R. Parikh

**Affiliations:** ^1^Department of Cellular and Molecular Medicine, Wilhelm Johannsen Centre for Functional Genome Research, Faculty of Health Sciences, Blegdamsvej 3B, University of Copenhagen, 2200 N, Copenhagen, Denmark; ^2^Department of Assisted Reproduction and Genetics, Jaslok Hospital and Research Centre 15, Dr. G Deshmukh Marg, Mumbai 400 026, India

## Abstract

Infertility is a complex human condition and is known to be caused by numerous factors including genetic alterations and abnormalities. Increasing evidence from studies has associated perturbed epigenetic mechanisms with spermatogenesis and infertility. However, there has been no consensus on whether one or a collective of these altered states is responsible for the onset of infertility. Epigenetic alterations involve changes in factors that regulate gene expression without altering the physical sequence of DNA. Understanding these altered epigenetic states at the genomic level along with higher order organisation of chromatin in genes associated with infertility and pericentromeric regions of chromosomes, particularly 9 and Y, could further identify causes of idiopathic infertility. Determining the association between DNA methylation, chromatin state, and noncoding RNAs with the phenotype could further determine what possible mechanisms are involved. This paper reviews certain mechanisms of epigenetic regulation with particular emphasis on their possible role in infertility.

## 1. Introduction

Identifying factors involved in the aetiology and physiology of complex disorders and conditions such as infertility is necessary in order to understand potential regulatory mechanisms involved in disease pathogenesis. Infertility has previously been defined as the inability to conceive after a passage of twelve months of unprotected intercourse by a couple [1, 2]. The condition has been estimated to have an effect on 10% of the population within the reproductive age group in the United States [2] and on 9% of the world's population [3]. The infertility phenotype affects both men and women and has been shown to have an impact on one's mental state and lifestyle and has also been implicated with being the cause or effect of certain medical conditions [1, 4]. The occurrence of such a high population incidence rate could be attributed to the afflicted individual's age, environment, and lifestyle. Studying the association of these factors in the part they play with genetic factors and altered epigenetic mechanisms regulating gene expression could facilitate a better understanding of this chronic condition [5, 6]. A deeper insight into the molecular genetics of complex disorders was revealed with the initial sequencing analysis of the human genome, which gave a wealth of information pertaining to the physical sequence of DNA but also provided significant details about the vast majority of the human genome that is non-protein coding [7–10]. Although it has been established that the whole human genome is transcribed at some point during the cell cycle [11, 12]; deciphering the role of the different molecular mechanisms involved in selective expression of protein coding and noncoding regions of the human genome at different time points of the cell cycle, in a tissue-specific manner during one's development, [13–15] in normal and diseased tissue, will further promote the understanding of complex human conditions and diseases, such as infertility. Several studies have previously identified gene deletions and polymorphisms associated with male factor infertility. Deletions in the Azoospermia Factor C (*AZFc*, OMIM #415000) region of the long arm of the Y chromosome proximal to the large heterochromatic block, including polymorphisms and deletions in the Ubiquitin-specific peptidase 9 and Y-linked (*USP9Y, *OMIM #400005) gene [16], have been identified as the most common cause of male factor infertility, particularly spermatogenic failure [17]. Mechanisms involved in the proper regulation of genomic and chromosomal variants [18, 19] associated with infertility in individuals having bad obstetric history (BOH) or repeated spontaneous abortions (RSA) and idiopathic cases of infertility remain largely unknown, although an understanding of the possible causes is beginning to emerge [20]. Understanding the complex role of one's genotype, environment, and age with changes in one's epigenotype could further categorise the unknown causes of the disease in addition to understanding the regulatory mechanisms involved in the control of expression and/or repression of genes affecting the infertility phenotype.

## 2. Regulatory Epigenetic Mechanisms

Waddington (1953) first described mechanisms associated with the alteration of gene expression in a cell during development as epigenetic [21]. An epigenetic change is defined as a heritable change that can alter the expression of a gene without actually changing the physical sequence of DNA [22]. Epigenetic mechanisms may control the expression of a gene via transient or permanent changes in its activity and are postulated to include three main processes [23]. These include reversible covalent modifications of histone core proteins, long and short noncoding RNA (ncRNA) related silencing of gene expression, and reversible methylation of DNA [24–26]. Each of these mechanisms has been associated with the initiation of the other [27] although the direction of control and regulation in different regions of the compartmentalised genome is under constant review [27–30]. Understanding the way these epigenetic marks regulate each other or induce recruitment of factors involved in the silencing or activation of gene expression in a locus-specific manner, in different regions of the genome, has yet to be determined. 

Epigenetic alterations or epimutations, particularly in the pattern of genomic DNA methylation including the 5′ promoter regions of genes, have been associated with various human conditions and disorders [23]. These include certain cancers [31], neurological disorders [26], abnormal sperm profiles in infertile men [20], the Rett syndrome [32] (RTT, OMIM# 312750), the Fragile X syndrome [33, 34] (FRAXA, OMIM# 309550), the ICF syndrome (facial anomalies) syndrome [35] (ICF, OMIM# 242860), Dihydropyrimidine dehydrogenase deficiency [36] (DPYD, OMIM# 274270), Prader-Willi syndrome (PWS, OMIM# 176270), and Angelman syndrome [37] (AS, OMIM# 105830). Methylation of CpG dinucleotides in the promoter regions of genes [38] and DNA methyltransferases (DNMTs) involved in the catalysis of DNA methylation [39–44], including 5-hydroxymethylcytosine [38, 45, 46] and altered covalent modifications of core histone proteins [47–49], have generated a great deal of interest in recent years. Tissue-specific patterns of DNA methylation in genes and various mechanisms of epigenetic control such as histone modifications and chromatin rearrangements have been associated with the regulation of genes and their expression or repression [13, 44, 50, 51]. Chromatin is not uniform in gene density and transcriptional activity. Selective expression of certain genes serving functionally important purposes at different stages of development is required in an organism to maintain tissue specificity [14, 52]. This process is done by compartmentalisation, which involves the packaging of certain parts of the genome into either actively transcribed euchromatin or transcriptionally silent/inert heterochromatin. Mechanisms involved in the maintenance of both silent heterochromatin and active euchromatin at different stages of the cell cycle in relation to nuclear organisation are beginning to be understood [51, 53]. 

The derepression of otherwise repressed genes has previously been reported and includes the activation/expression of the sperm genome in the embryo [54], viral oncogenesis, and activation of Y chromosome genes during the foetal development of a male child [55]. The multistep process of X chromosome inactivation (XCI) induced by the coordinated active transcription of the long noncoding RNA *Xist* and *Tsix* [56] involves the random transcriptional inactivation of either one of the X chromosomes in female mammals [57] in response to certain cellular stimuli. This coordinated upregulation of the *Xist *transcript compared to the downregulation of *Tsix* on the inactive X chromosome (Xi) of future daughter cells equalizes the expression of X linked genes in female (XX) and male (XY) embryos [56, 58], a process known as dosage compensation [59]. Higher order structures such as euchromatin and heterochromatin represent the degree of packaging of genomic DNA thereby rendering it either accessible or inaccessible to transcription machinery. Particular modifications of core histones H2A, H2B, H3, and H4 that form the nucleosome and their variants including H2A.Z, H3.1, H3.2, and H3.3 are seen to be conserved in certain organisms [57, 60]. Mechanisms involved in covalently altering these histone modification marks particularly on histone tails, include phosphorylation, acetylation, ubiquitination, and methylation [48]. Histone tails have previously been linked with playing an essential role in conformational changes associated with histone folding and higher order chromatin packaging [61]. Unlike the globular core histone domains, histone tails do not have fixed structures and can be modified by posttranslational mechanisms [62]. Covalent modifications of histone tails altering their conformation and charge allow for potential activation of transcription (hyperacetylated histone tails, histone 3 lysine 4 trimethylated) or repression (hypoacetylated histone tails histone 3 lysine 27 trimethylated) of the packaged DNA by the recruitment of certain protein complexes [63] at different stages of cell cycle progression. Differential chromatin modification marks have been recently associated with pericentric heterochromatin and the inactive X chromosome [28, 64]. A study carried out by Maison et al. attributes the formation of higher order pericentromeric heterochromatin to a specific RNA component and distinct covalent histone modification marks that are unique to this region compared to the inactive X chromosome [28]. This study implicated the presence of an RNA component with the initiation, formation, and maintenance of pericentromeric heterochromatin, identified by the presence of heterochromatin protein 1 (HP1) and histone 3 lysine 9 (H3K9) marks [28]. Another study carried out by Chen et al. (2008) looked at the dynamic nature of centromeric heterochromatin in the model organism *Schizosaccharomyces pombe* and implicated it with being actively transcribed by RNA Pol II, albeit during a brief period of the cell cycle. They examined this brief period of the cell cycle during the S-phase and found that RNA Pol II is involved in transcribing centromeric repeats [65]. They implicated the maintenance of heterochromatin with that of RNA Pol II mediated transcription of repeat rich regions and silencing of the same region with the involvement of factors recruited by the histone 3 lysine 9 (H3K9) modification mark [65]. This negative feedback loop associated in both studies could possibly be associated with the proper maintenance of heterochromatin including centromeric heterochromatin through subsequent cell divisions allowing for proper mitotic cell division, mechanistically similar to X chromosome inactivation, albeit involving different factors. Variations and differences in higher order structural organisation of this late replicating region of certain chromosomes particularly heterochromatic blocks on chromosomes 9 and Y have previously been associated with infertility [19, 66, 67]. Although experimentally challenging, understanding the molecular epigenetic and regulatory landscapes of these repeat rich regions in normal individuals compared to those with infertility and deciphering their altered epigenetic signature, if any, would result in a broader comprehension of depicting reproductive outcome.

## 3. Factors Associated with the Infertility Phenotype

Several studies have identified different genetic and epigenetic factors as being involved with the onset and progression of the infertility phenotype [17, 19, 68]. However, possible perturbed mechanisms involved in regulating gene expression in the disease state are rather poorly understood. The classic “nature versus nurture” argument on whether one's environment rather than genetic makeup could influence the onset of infertility and other complex disorders has been reviewed by various studies [69, 70].

## 4. Environment and the Infertility Phenotype

Kohler et al. (1999) studied the presence of the infertility phenotype in a cohort of Danish twins to be able to answer two fundamental questions in relation to infertility, whether there was a significant effect on an individual being infertile due to his/her genetic predisposition or his/her environment. While the study concluded that genetic factors do play a part in infertility, it also raised the possibility of a connection between infertility and one's socioeconomic status and particularly the environment [70]. Another study carried out by Cloonan et al. (2007) examined the existence of male factor infertility in a Vietnamese male twin cohort [69]. The authors concluded that the environment or genetic makeup of individual twins did not have a significant effect on any one twin having infertility, but that conditions and factors unique to individual twins could be associated with the disorder, although it did not rule out an indirect effect of one's environment on these factor [69]. This further established a link between epigenetic mechanisms unique to each individual and the onset of infertility. Both these studies agreed on factors unique to each individual twin as being associated with the disease phenotype. These epigenetic factors including DNA methylation and chromatin state, unique to each monozygotic twin could be attributed in part to their infertile state.

## 5. Epigenetic Factors Influencing the Infertility Phenotype

Several histone de/acetylases and demethylases have recently been identified and attributed with regulation of chromatin state. However, their functional role and association with diseased states is only just beginning to be understood, particularly those factors affecting male and female infertility [71, 72]. Post translational modifications of histone tails by factors including histone chaperones and methyltransferases are involved in the proper regulation of gene expression [73, 74]. Due its dynamic nature and plasticity, the landscape of chromatin can be altered, rendering a region of the genome active or inactive [74–76]. This altered state of packaging renders certain regions of the genome more accessible to transcription machinery (euchromatin) and are marked by DNA hypomethylation, RNA Pol II, and covalent histone modifications such as histone 3 trimethylated at lysine 4 (H3K4me3) and the histone variant H2A.Z. Inactive/repressed regions are known to be associated with DNA hypermethylation, histone 3 trimethylated at lysine 27 (H3K27me3), and SUZ12 (part of the polycomb group complex, PcG) [48]. 

Due to this, the main focus of recent epigenetic research has focussed on discovering new factors involved in altering chromatin state and further looking at its involvement in diseased and normal tissue. Recent studies have identified a critical role for the JHMD2A (Jumonji C domain-containing histone demethylase 2A) histone demthylase in male infertility, obesity [72], and spermatogenesis [77]. Using knock-out mice as models, these studies identified a critical role for JHMD2A in the regulation and expression of two genes, protamine 1 (OMIM #182880, *Prm1*) and transition nuclear protein 1 (OMIM #190231, *Tnp1*) involved in the condensation and proper packaging of chromatin in the male sperm [77]. A higher degree of sperm DNA compaction has previously been attributed to the increased presence of highly basic protamine proteins compared to histones in chromatin [78], a deficiency of which has been associated with infertility in mice [79, 80]. Identification of other regulatory mechanisms involved in the recruitment of factors, in addition to JHMD2A, involved in the deposition of histones along with others affecting transcriptional activity of genes involved in infertility will increase our understanding of mechanisms involved in both perturbed and normal states.

## 6. Epigenetic Modifications Affecting Reproductive Outcome

Imprinted regions of the human genome marked by one active and one inactive allele are known to be dependent on their parent of origin [25, 81]. These monoallelically expressed genes are known to be significantly associated with fetal development, reproduction, and reproductive outcome [82, 83]. Deregulation of imprinted regions associated with the onset of Angelman syndrome has previously been document in cases undergoing ICSI (intracytoplasmic sperm injection) using assisted reproductive technologies (ART) [84, 85]. Reprogramming of the epigenome and imprinted loci during gametogenisis and the preimplantation embryonic stage is essential for maintaining the pattern of proper inheritance, specifically at imprinted loci [82]. Deregulation of imprinted loci has previously been associated with malformed offspring including disruption of the *Igf2* imprinted region in mice. Disruption of this imprinted region in mice results in their offspring being retarded [86] and loss of imprinting (LOI) of this same region results in them having Beckwith-Wiedemann syndrome (BWS) [87]. Long-term cohort studies looking at the incidence of imprinting disorders and the use of ART have failed to draw a significant relation between the two [88, 89]. Identifying mechanisms associated with the regulation of imprinted loci could further help in understanding their role in proper parental inheritance of expression pattern of imprinted genes and their possible perturbed state associated with the infertility phenotype.

## 7. Conclusions and Future Prospects

Studies facilitating the identification of factors involved in the proper maintenance and organisation of repeat rich pericentromeric heterochromatic regions could be important in understanding their association with their higher occurrence rate in certain infertile groups [19, 90]. Results from corelated epidemiological studies [91, 92] along with factors such as one's environment, age, epigenotype, and genotype could provide a greater understanding of how different gene regulatory pathways are controlled and affect each other [6] in human diseases like infertility [20]. 

Deciphering which epigenetic mechanism/s, if any, are altered in certain infertile subjects with increased pericentromeric blocks of heterochromatin on chromosomes 9 and Y, both locally or globally would help further characterise the disorder at both molecular genetic and epigenetic levels. Identifying the degree to which an altered epigenetic state can affect the development of infertility remains largely unknown although studies using newer technologies are now able to question and understand the potential mechanisms involved. Although the study of epigenetic factors affecting infertility is at its nascent stage, a clearer picture is beginning to emerge that is helping in the identification of new factors involved. The further development of methods to advance our understanding of regulatory control mechanisms of genes affecting human infertility and reproductive outcome in addition to what has been done so far could assist in improving rates of pregnancy using assisted reproductive techniques and provide better treatment options for individuals seeing treatment for infertility or subfertility.
